# Preoperative Recognition of Vertebral Number Variations in Adolescent Idiopathic Scoliosis: A Series of Four Cases

**DOI:** 10.7759/cureus.115425

**Published:** 2026-08-30

**Authors:** Asim Abdelgader A Farah, Rana Alrasheed, Waqar Hassan

**Affiliations:** 1 Orthopedic Surgery, Qassim Health Cluster, Buraydah, SAU

**Keywords:** adolescent idiopathic scoliosis, fusion level selection, lumbar vertebrae number variation, thoracic vertebrae number variation, wrong-level spine surgery

## Abstract

Numerical variations in thoracic and lumbar vertebrae in adolescent idiopathic scoliosis (AIS) are not uncommon but are often underrecognized. This case series highlights the impact of identifying these variations on operative planning and correct fusion level identification.

We report four patients with AIS with thoracic and lumbar vertebral number variations that were identified on standing whole-spine radiographs. Vertebral counts, curve characteristics, Lenke classification, and planned fusion levels are presented to illustrate the impact of these variations on surgical planning and approach.

Increasing surgeons' awareness of the importance of vertebral enumeration is essential to ensure accurate documentation and fusion level selection and to avoid unnecessary costs, prolonged operative time, and associated complications.

## Introduction

Variations in vertebral number, such as the presence of 11 or 13 thoracic vertebrae or 4 or 6 lumbar vertebrae, as well as cervical ribs and lumbosacral transitional vertebrae (LSTV), may influence accurate vertebral level identification. In adolescent idiopathic scoliosis (AIS), where long fusion constructs are often required, alterations in vertebral numbering may influence surgical planning and fusion level selection. Therefore, routine identification and documentation of vertebral levels are essential for safe scoliosis surgery.

Previous studies have reported that approximately 10-20% of patients with AIS have numerical vertebral variations, many of which may remain unrecognized without intentional vertebral counting [1,2]. Importantly, these variations are often underreported in radiological reports.

Higher prevalence rates have been described in specific populations, and large cohorts have demonstrated that atypical thoracic-lumbar vertebral distributions may exist even when the total vertebral count appears normal [3,4]. These findings suggest that reliance on assumed vertebral anatomy remains a potential source of surgical error.

Most published studies are based on large retrospective cohorts; however, detailed case-level descriptions highlighting how variations in vertebral number directly affect surgical planning remain limited. This case series aims to provide practical clinical examples that highlight diagnostic pitfalls and preventive strategies.

## Case presentation

Case 1

A 17-year-old female with AIS presented for evaluation. Standing whole-spine posteroanterior (PA) radiographs showed an atypical thoracic vertebral count with 11 thoracic vertebrae (Figure 1A). Cobb angles measured 60° from T4 to T10 and 55° from T11 to L5, consistent with Lenke classification type 3BN. Posterior spinal fusion and deformity correction were performed from T3 to L3 (Figure 1B). 

**Figure 1:**
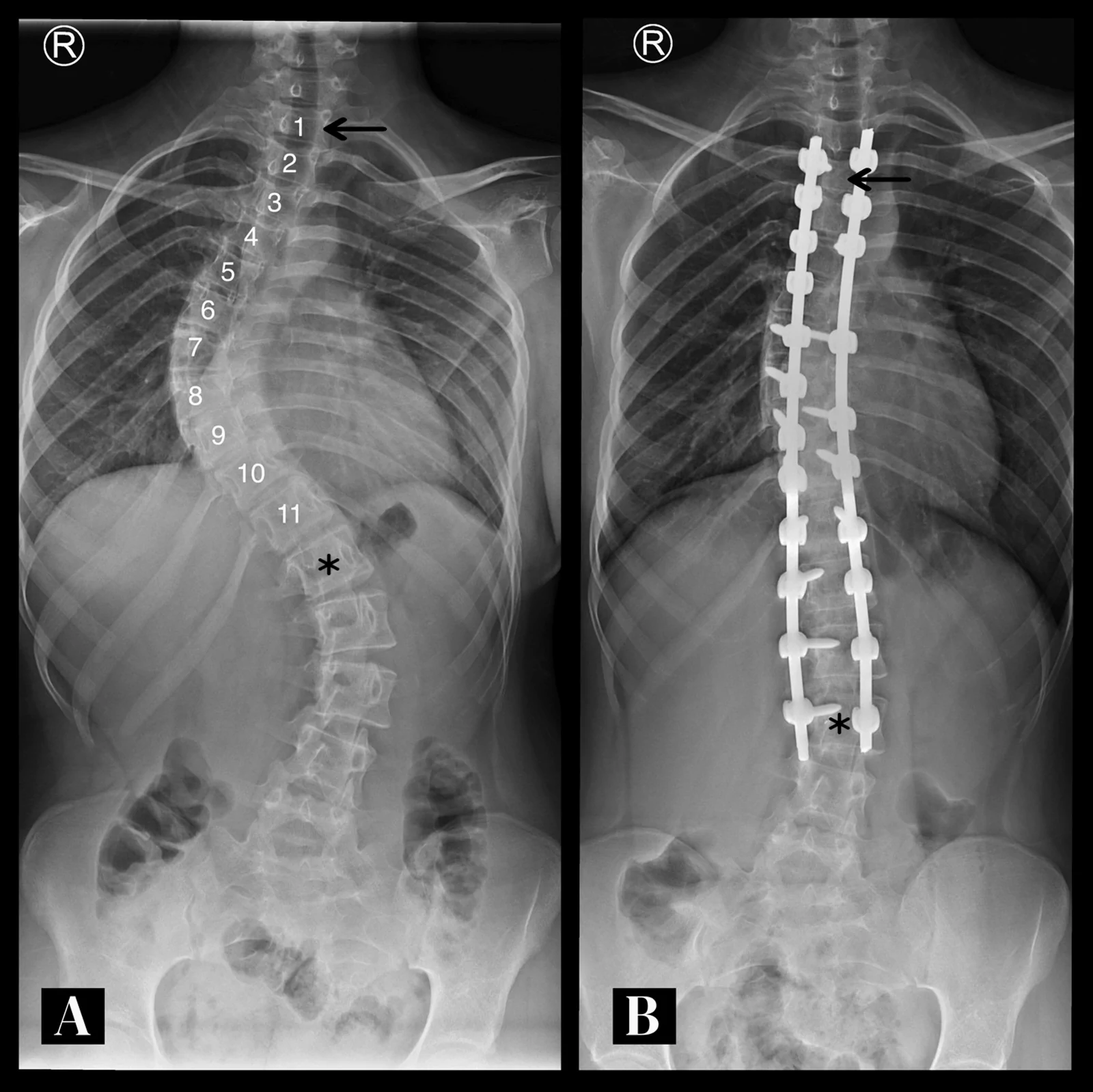
Preoperative (A) and postoperative (B) whole spine posteroanterior (PA) radiograph. (A) The preoperative radiograph shows 11 thoracic vertebrae, counted from the first rib-bearing vertebra (black arrow), and 5 lumbar vertebrae, counted from the first non-rib-bearing vertebra (black asterisk). (B) The postoperative radiograph shows posterior spinal deformity correction and fusion from T3 (black arrow) to L3 (black asterisk).

Case 2

A 17-year-old female with AIS presented for evaluation. Standing whole-spine PA radiographs showed an atypical lumbar vertebral count with 4 lumbar vertebrae (Figure 2A). The major curve measured 62° from T6 to L1, consistent with Lenke classification type 1BN. Posterior spinal fusion and deformity correction were performed from T3 to L1 (Figure 2B). 

**Figure 2:**
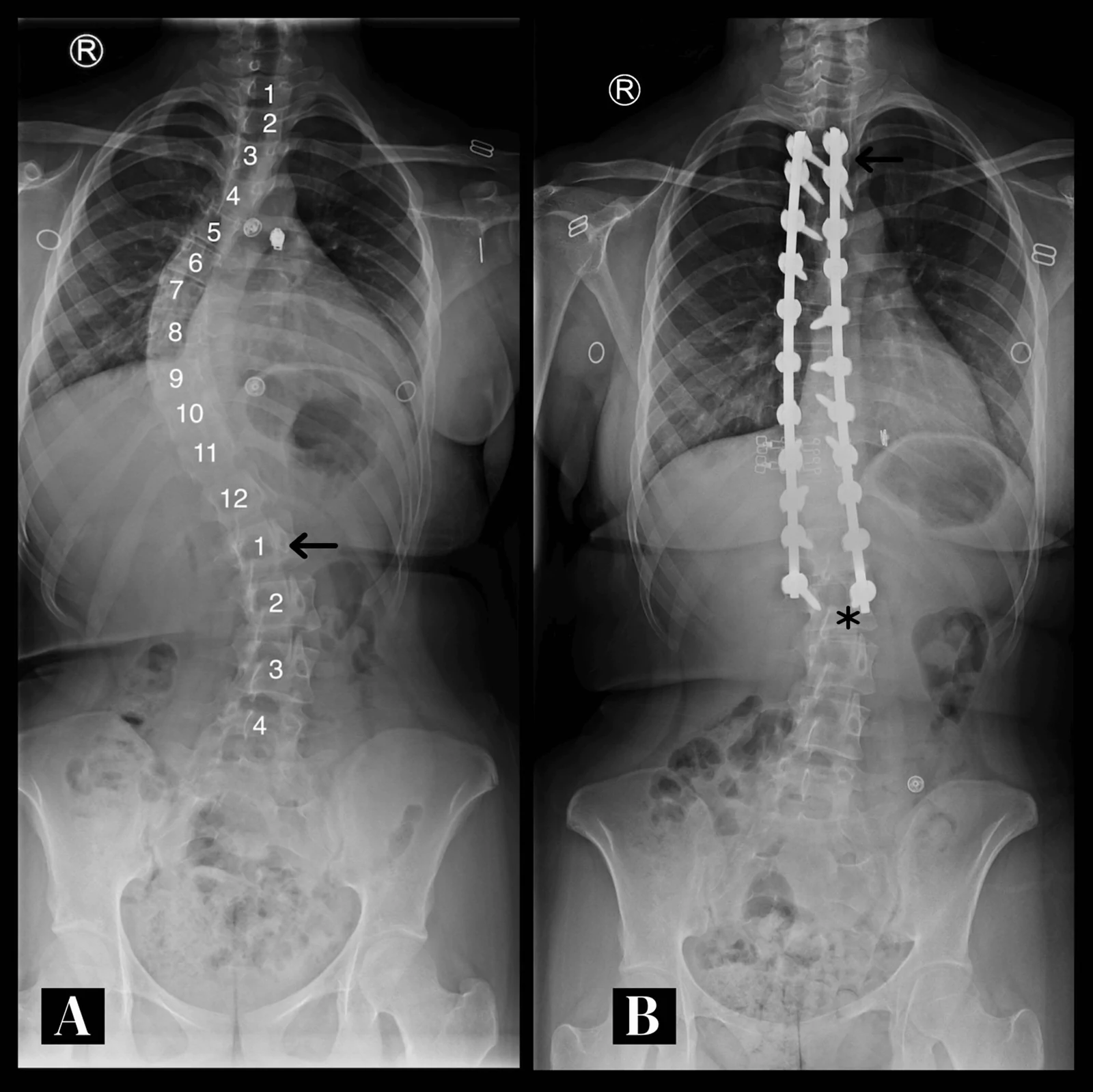
Preoperative (A) and postoperative (B) whole spine posteroanterior (PA) radiograph. (A) The preoperative radiograph shows 4 lumbar vertebrae, counted from the first non-rib-bearing vertebra (black arrow). (B) The postoperative radiograph shows posterior spinal deformity correction and fusion from T3 (black arrow) to L1 (black asterisk).

Case 3

A 20-year-old male with AIS presented for evaluation. Standing whole-spine anteroposterior (AP) radiographs showed an atypical thoracic vertebral count with 11 thoracic vertebrae (Figure 3A). The major curve measured 63° from T5 to L1, consistent with Lenke classification type 1BN. Posterior spinal fusion and deformity correction were performed from T3 to L2 (Figure 3B). 

**Figure 3:**
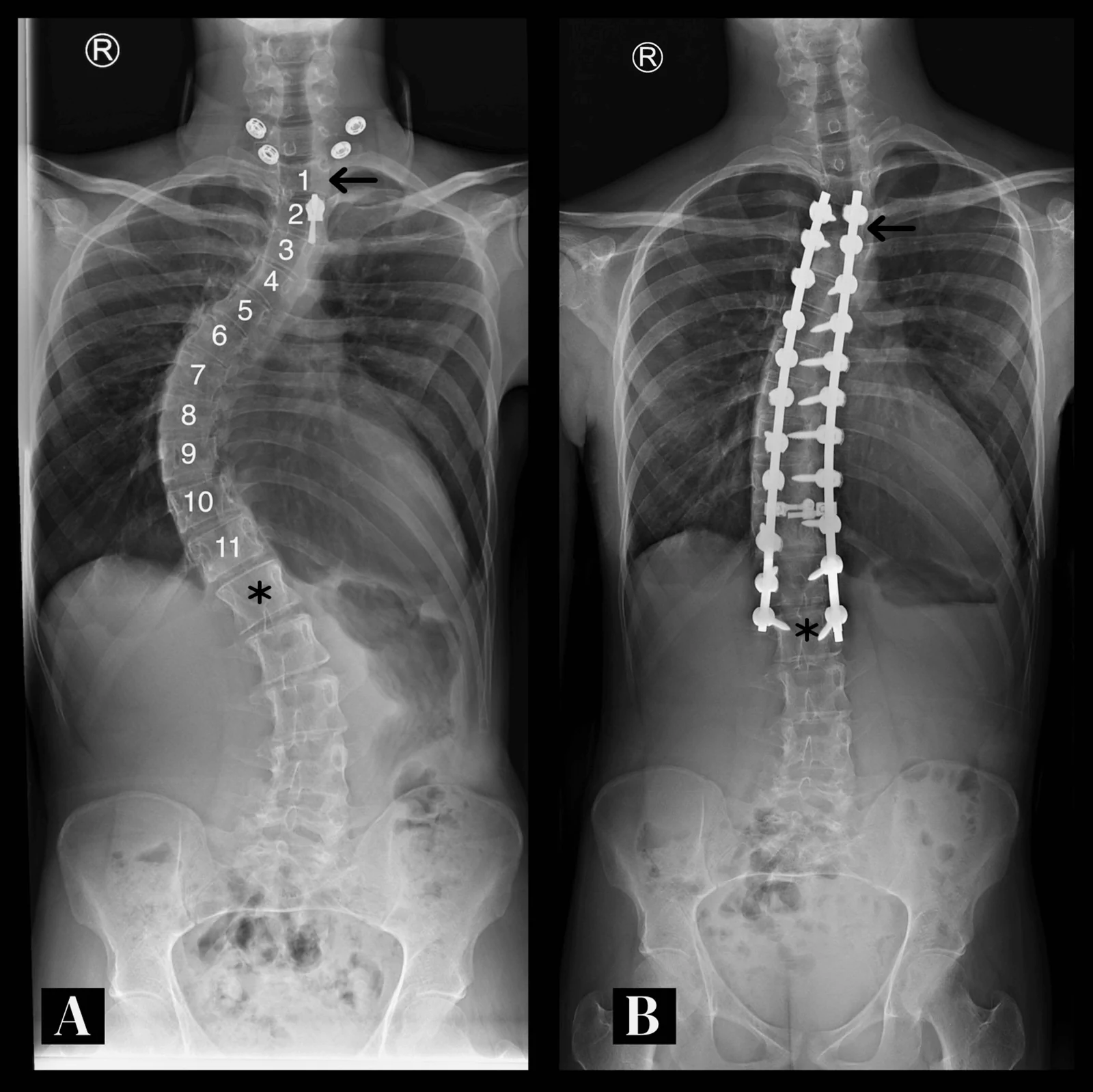
Preoperative (A) and postoperative (B) whole spine posteroanterior (PA) radiograph. (A) The preoperative radiograph shows 11 thoracic vertebrae, counted from the first rib-bearing vertebra (black arrow), and 5 lumbar vertebrae, counted from the first non-rib-bearing vertebra (black asterisk). (B) The postoperative radiograph shows posterior spinal deformity correction and fusion from T3 (black arrow) to L2 (black asterisk).

Case 4

A 17-year-old female with AIS presented for evaluation. Standing whole-spine PA radiographs showed an atypical thoracic vertebral count with 11 thoracic vertebrae (Figure 4A). The major curve measured 52° from T5 to T11, consistent with Lenke classification type 1BN. Posterior spinal fusion and deformity correction were performed from T4 to L2 (Figure 4B). 

**Figure 4:**
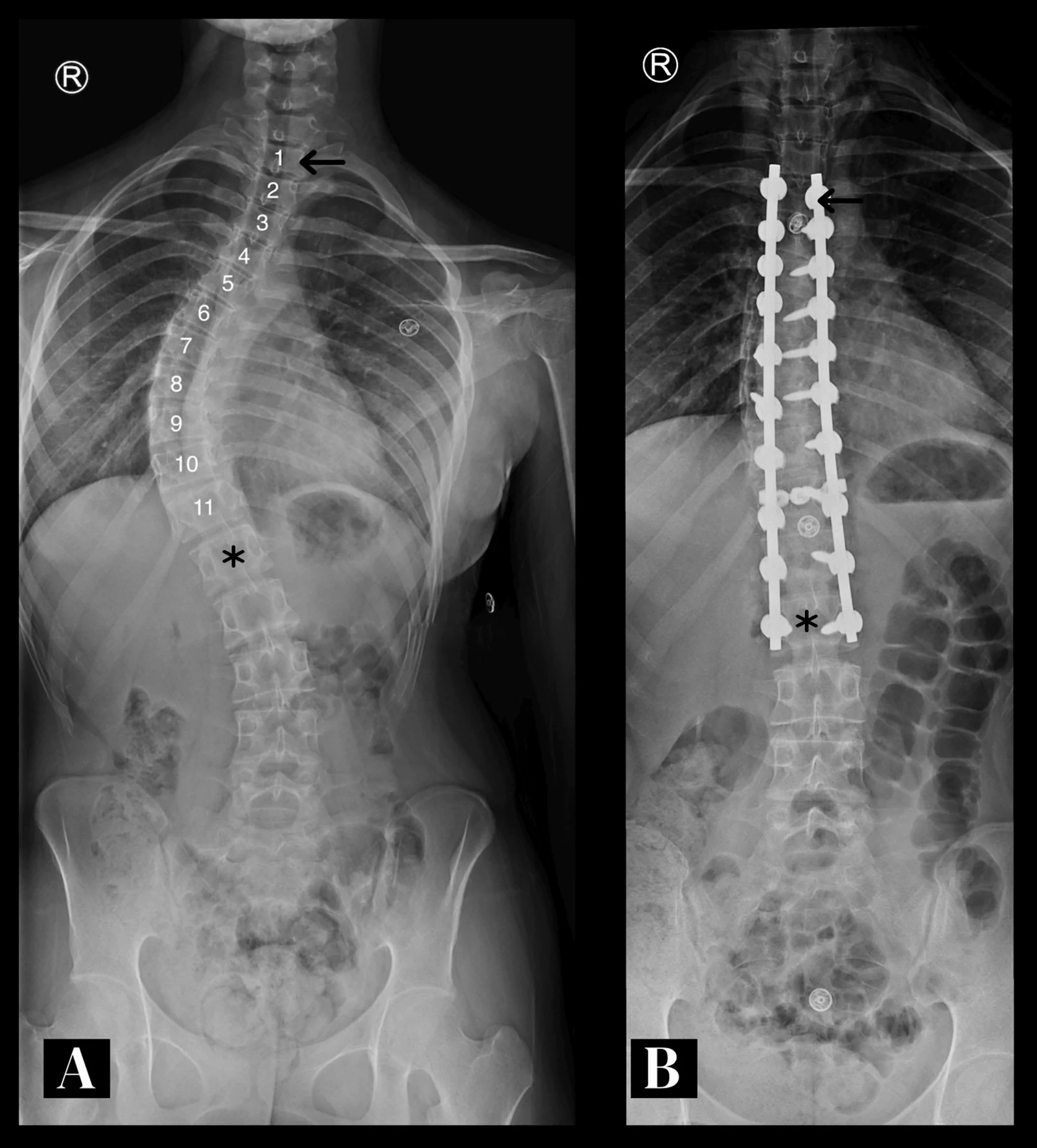
Preoperative (A) and postoperative (B) whole spine posteroanterior (PA) radiograph. (A) The preoperative radiograph shows 11 thoracic vertebrae, counted from the first rib-bearing vertebra (black arrow), and 5 lumbar vertebrae, counted from the first non-rib-bearing vertebra (black asterisk). (B) The postoperative radiograph shows posterior spinal deformity correction and fusion from T4 (black arrow) to L2 (black asterisk).

Patient selection, surgical planning, and intraoperative level localization 

We retrospectively reviewed all consecutive patients with AIS (20 patients) who underwent corrective surgery between October 2024 and October 2025 at our institution. Four patients with vertebral number variations identified during preoperative planning were included. Demographic data, imaging findings, vertebral enumeration, fusion levels, and surgical outcomes were reviewed. The characteristics of the four cases are summarized in Table 1. 

**Table 1:** Clinical, radiographic, and surgical characteristics of the presented cases. Major curves were measured on standing whole-spine radiographs. Thoracic vertebral levels were identified using rib-based enumeration caudally from C2 to the sacrum.

Case	Age (Years)/Sex	Presacral Vertebral Count	Major Curve(s) (Cobb Angle, Degree)	Lenke Type	Planned Fusion Levels	Number of Instrumented Vertebrae
1	17/Female	23 vertebrae: 7 cervical, 11 thoracic, 5 lumbar	Thoracic (T4-T10): 60° Lumbar (T11-L5): 55°	3BN	T3-L3	12 vertebrae
2	17/Female	23 vertebrae: 7 cervical, 12 thoracic, 4 lumbar	Main thoracic (T6-L1): 62°	1BN	T3-L1	11 vertebrae
3	20/Male	23 vertebrae: 7 cervical, 11 thoracic, 5 lumbar	Main thoracic (T5-L1): 63°	1BN	T3-L2	11 vertebrae
4	17/Female	23 vertebrae: 7 cervical, 11 thoracic, 5 lumbar	Main thoracic (T5-T11): 52°	1BN	T4-L2	10 vertebrae

Vertebral numbering was performed using different methods. On whole-spine standing radiographs, vertebrae were counted caudally from C2 to the sacrum. Rib-bearing vertebrae were classified as thoracic. The total presacral vertebral count was recorded for each patient. Computed tomography (CT) was used when additional anatomical clarification was required. The CT scans obtained helped confirm the thoracic vertebral count and exclude associated vertebral anomalies in all cases except Case 2, in which CT imaging was not performed because it was not clinically indicated.

In all cases, intraoperative level identification was performed using intraoperative X-ray. The lower instrumented vertebra (LIV) was identified with reference to L5, except in Case 2, in which L4 was used as the reference level. During surgical exposure, vertebral levels were counted cranially by direct visualization to identify the upper instrumented vertebra (UIV), with final confirmation obtained by referencing T1 on intraoperative X-ray.

## Discussion

Anatomical variation in AIS is not uncommon. Prior studies have reported a prevalence of approximately 10% for variations in vertebral number in AIS, with atypical lumbar anatomy occurring more frequently and LSTV present in up to 25% of affected patients [2]. Despite this, such variations are markedly underreported in radiology reports [2]. Comparable prevalence rates of 10.6-10.9% have been demonstrated across different populations, with a higher frequency observed in males and a male-to-female ratio of approximately 1.2:1 [1].

Although the prevalence of wrong-level surgery in AIS has not been directly studied, prior investigations have shown that up to 50% of spine surgeons report having performed wrong-level surgery during their careers [5]. Atypical spinal anatomy and failure to verify the operative level radiographically have been identified as major contributing factors [5]. Given this, failure to recognize these anatomical variations may theoretically result in incorrect fusion level selection, including unintended proximal or distal extension of instrumentation, fusion across unnecessary segments, or termination of the fusion at a transitional vertebra. Such errors may adversely affect shoulder balance, compromise curve correction, increase the risk of implant-related complications, and reduce distal segmental mobility. In addition, unnecessary extension of fusion constructs may contribute to increased operative time, morbidity, and healthcare costs.

In our case series, we investigated the potential relationship between variations in vertebral number and their impact on operative planning and fusion level selection. None of the vertebral variations in our cases were reported by the radiologist. All variations were identified by the surgeons through evaluation of whole-spine radiographs. Previous studies have also emphasized that radiological evaluation should be performed by the surgeon as part of the preoperative assessment rather than relying solely on radiology reports [6,7].

In the present series, three patients had 11 thoracic vertebrae, and one patient had 4 lumbar vertebrae. No cervical ribs or LSTV were identified. Vertebral enumeration was performed using whole-spine plain radiographs. Vertebral counting was performed using different approaches, including caudal enumeration from C2 to the sacrum on whole-spine standing radiographs. Rib-bearing vertebrae were classified as thoracic vertebrae, and the total presacral vertebral count was recorded for each patient. The cases with 11 thoracic vertebrae also had preoperative CT scans, which aided in confirming these variations; however, the variations were identifiable on plain radiographs alone. In the second case, CT was not obtained because it was not clinically indicated; therefore, CT confirmation regarding the possibility of an LSTV was not available, and vertebral enumeration relied solely on plain radiographs. Previous work has demonstrated that cephalocaudal enumeration on whole-spine imaging is superior to MRI for identifying LSTV [8].

Regarding fusion level selection, particularly in Case 1, the LIV was selected primarily based on the stable vertebra and touched vertebra criteria, together with the overall coronal alignment and flexibility of the lumbar curve. Although the curve anatomically extended to L5, the preoperative assessment supported stopping the fusion at L3, and this decision was further confirmed intraoperatively. Importantly, the presence of vertebral numerical variation did not alter the selected fusion levels in any of the cases. However, we would like to highlight that, in patients with 11 thoracic vertebrae, the total number of instrumented vertebrae may be reduced by one compared with patients with the typical vertebral count, depending on the selected fusion levels. In contrast, the presence of 4 lumbar vertebrae in our series did not affect the total number of instrumented vertebrae.

Although no incorrect fusion level selection occurred in our cases, failure to recognize vertebral numerical variations preoperatively and reliance solely on identification of the LIV or UIV and direct intraoperative visualization may theoretically increase the risk of incorrect level selection. Accurate vertebral enumeration before surgery is therefore important to ensure that the intended anatomical levels correspond to the planned fusion levels.

The purpose of this study was to highlight the potential implications of individual anatomical variations for fusion level selection in AIS. Emphasizing surgeon-led vertebral enumeration during preoperative planning is critical to ensure accurate vertebral level documentation, appropriate fusion strategy, and avoidance of the theoretical risk of wrong-level fixation.

## Conclusions

Numerical variations in the thoracic and lumbar vertebral count are not uncommon in AIS and may have implications for vertebral level identification and surgical planning. This case series highlights the importance of systematic preoperative vertebral enumeration and accurate documentation of vertebral numbering, particularly in patients with atypical vertebral anatomy. Although vertebral numerical variations did not alter fusion level selection in our cases, failure to recognize such variations may theoretically increase the risk of incorrect level identification. Surgeon-led vertebral enumeration as part of preoperative planning may therefore help ensure accurate vertebral level identification and reduce the theoretical risk of wrong-level fixation. Larger studies are needed to determine the clinical impact of vertebral numerical variations and to assess the actual incidence of wrong-level surgery in this population.

## References

[1] Hu Z, Zhang Z, Zhao Z, Zhu Z, Liu Z, Qiu Y (2016). A neglected point in surgical treatment of adolescent idiopathic scoliosis: variations in the number of vertebrae. Medicine (Baltimore).

[2] Ibrahim DA, Myung KS, Skaggs DL (2013). Ten percent of patients with adolescent idiopathic scoliosis have variations in the number of thoracic or lumbar vertebrae. J Bone Joint Surg Am.

[3] Garg B, Mehta N, Goyal A, Rangaswamy N, Upadhayay A (2021). Variations in the number of thoracic and lumbar vertebrae in patients with adolescent idiopathic scoliosis: a retrospective, observational study. Int J Spine Surg.

[4] Chiu CK, Chin TF, Chung WH, Chan CY, Kwan MK (2024). Variations in the number of vertebrae, prevalence of lumbosacral transitional vertebra and prevalence of cervical rib among surgical patients with adolescent idiopathic scoliosis: an analysis of 998 radiographs. Spine (Phila Pa 1976).

[5] Mody MG, Nourbakhsh A, Stahl DL, Gibbs M, Alfawareh M, Garges KJ (2008). The prevalence of wrong level surgery among spine surgeons. Spine (Phila Pa 1976).

[6] Clark R, Anderson MB, Johnson BH, Moore DE, Herbert FD (1996). Clinical value of radiologists' interpretations of perioperative radiographs of orthopedic patients. Orthopedics.

[7] Crockett HC, Wright JM, Burke S, Boachie-Adjei O (1999). Idiopathic scoliosis. The clinical value of radiologists' interpretation of pre- and postoperative radiographs with interobserver and interdisciplinary variability. Spine (Phila Pa 1976).

[8] O'Driscoll CM, Irwin A, Saifuddin A (1996). Variations in morphology of the lumbosacral junction on sagittal MRI: correlation with plain radiography. Skeletal Radiol.

